# Physical Characteristics and Microbial Quality of Ostrich Meat in Relation to the Type of Packaging and Refrigerator Storage Time

**DOI:** 10.3390/molecules26113445

**Published:** 2021-06-06

**Authors:** Olaf K. Horbańczuk, Artur Jóźwik, Jarosław Wyrwisz, Joanna Marchewka, Agnieszka Wierzbicka

**Affiliations:** 1 Department of Technique and Food Product Development, Institute of Human Nutrition Sciences, Warsaw University of Life Sciences (WULS-SGGW), 159c Nowoursynowska, 02-776 Warsaw, Poland; jaroslaw.wywrisz@sggw.edu.pl (J.W.); agnieszka_wierzbicka@sggw.edu.pl (A.W.); 2 Institute of Genetics and Animal Biotechnology, Polish Academy of Sciences, 05-552 Jastrzębiec, Poland; aa.jozwik@igbzpan.pl (A.J.); j.marchewka@igbzpan.pl (J.M.)

**Keywords:** ostrich meat, microbial quality, color, pH, cooking loss, drip loss, shear force

## Abstract

The aim of this study was to evaluate the effect of the packaging system type on the physical characteristics and microbial changes in ostrich meat during refrigerated storage. The applied packaging systems were vacuum packaging (VP) and modified atmosphere packaging (MAP) using two combinations of gases: MAP1 (40% O_2_/40% CO_2_/20% N_2_) and MAP2 (60% O_2_/30% CO_2_/10% N_2_). Eight meat samples were obtained in three replicates for all parameters, except for pH, for which six replicates were obtained from the *M. ilifibularis* (IF) muscle, and were stored in a refrigerator at 2 °C and analyzed at 0, 4, 8, 12 and 16 days for the effect of packaging methods on physical meat quality. The initial pH (5.99) decreased at the end of the storage time for MAP1 to 5.81, whereas VP was stable from day 0 to 12 and increased up to 6.08 on day 16. Regarding meat color, the L* value increased during storage for MAP1 and MAP2 from 36.99 to 40.75 and 41.60, respectively, whereas it declined for VP to 34.22. The same tendencies were reported for redness (a*) and yellowness (b*). Drip loss was the lowest in MAP1 and highest in VP. The lowest total viable bacteria counts were identified in VP, as compared to MAP1 and MAP2.

## 1. Introduction

Ostrich meat is recognized as a dietetic product with high nutritive value [1,2,3] and is becoming increasingly popular, not only in South Africa but also in Asia, South America, North America, and Europe [4,5,6,7,8,9,10]. Among European countries, Poland is one of the leaders in production of ostrich meat, exporting ca. 500 tons per year [11]. This meat, dark red in color, is characterized by higher pH (about 6) as compared to beef or pork [12,13,14]. The relatively high pH value of ostrich meat negatively affects the quality of this meat during storage [15,16,17,18]. In retail, meat is most often packed in vacuum and modified atmosphere packaging. Vacuum packaging provides anaerobic conditions inside the package, which leads to shelf-life extension and provides stable color [19,20]. MAP prolongs shelf life with aerobic packaging conditions and results in a more attractive bright red color due to myoglobin oxygenation [21]. Extended shelf life and meat safety aspects are very important both for the meat industry and for the consumers [20]. However, until now, investigation on ostrich meat quality, shelf life, packaging type and storage is still limited. Thus, the aim of the study was to assess the changes in the physical characteristics and microbial quality of ostrich meat packed in vacuum (VP) and modified atmosphere (MAP) using two combinations of gases, O_2_:CO_2_:N_2_, i.e., 40:40:20 (MAP 1) and 60:30:10 (MAP 2), during refrigerated storage up to 16 days.

## 2. Results and Discussion 

### 2.1. pH

The change in pH value as related to the storage time and type of packaging are presented in Table 1. In the case of the modified atmosphere (MAP1) packaging system using a combination of gases (O_2_:CO_2_:N_2_, i.e., 40:40:20), the initial pH (5.99) on day 0 decreased at the end of storage time (on 16 day) to 5.81 (*p* ≤ 0.05). The pH value was lower in MAP1 (*p* ≤ 0.05) as compared to vacuum packaging on days 8, 12 and 16 (Table 1). A significant decrease in the pH value occurred in the samples stored in the MAP1 system (*p* ≤ 0.05), probably due to the higher concentration of CO_2_ (40%) in the package. CO_2_, by dissolving in the aqueous phase of meat, builds carbonic acid, which lowers the pH of meat [22]. In vacuum packaging (VP), pH was stable between day 0 to 12, whereas it increased on the 16th day of storage up to 6.08 (*p* ≤ 0.05). The increase in pH may be due to advanced proteolysis occurring in the vacuum-packed long-stored meat [23]. Proteins in meat with higher pH value have a higher water binding capacity, which could lead to a lower level of free water in the spaces between swelling muscle fibers [24].

It should be noted that Fernandez-Lopez et al. [15], who carried out research on ostrich steaks stored in four different packaging types: air exposure, vacuum and two different modified atmosphere packages (MAP: 80% CO_2_ + 20% N_2_ and MAP + CO: 30% CO_2_ + 69.8% argon + 0.2% CO), demonstrated a decline in pH in all types of packaging during storage time. Results similar to this study for vacuum-packed ostrich meat were obtained by Thomas et al. [25], who stored this meat up to 12 days at 4 °C.

### 2.2. Color Parameters

Overall, the L* (lightness) increased (*p* ≤ 0.05) during the storage for MAP1 and MAP2 (from 36.99 to 40.75 and 41.60, respectively), while for VP it decreased from day 12 onwards (34.22; *p* ≤ 0.05). The L* value was significantly higher (*p* ≤ 0.05) in ostrich samples packed in the MAP1 and MAP2 systems from the eighth day of storage as compared to VP (Table 1).

The redness of the investigated ostrich muscles in MAP1 and MAP2 increased significantly (*p* ≤ 0.05) on the fourth day of storage, likely due to myoglobin being converted into oxymyoglobin form. However, further storage caused a decrease in redness, which may be associated with a relatively high concentration of O_2_, which leads to oxidation of oxymyoglobin (formed up to day 4) into metmyoglobin; this results in a lower redness (lower a*) [26]. The value of b* was the lowest in the vacuum-packaging system compared to MAP2. Changes in muscle color during storage might be explained by the color of muscle tissue being conditioned by reflectance light of free water and the degree of oxidation of myoglobin [27]. A comparable trend of L* was found by Leygonie et al. [28] in stored frozen vacuum-packed ostrich meat, but the a* and b* values were almost on the same level. Seydim et al. [16], in their research on the effect of packaging on shelf-life quality of ground ostrich meat, stated that in vacuum packages the meat was darker (lower L*) as compared to 80% of O_2_ in MAP packages (higher L*). Similar changes in the a* and b* values in beef steaks packed in vacuum and MAP stored at 2 °C were reported by Łopacka et al. [29]. Filgueras et al. [30], in a study conducted on frozen vacuum-packed rhea meat stored for 180 days at −80 °C, reported a decline in L*, a* and b* coordinates. 

### 2.3. Drip Loss

The drip loss (%) of ostrich meat is shown in Table 2. Significant differences in drip loss (*p* ≤ 0.05) depending on the type of packaging and storage time were observed. In all packaging systems, the drip loss steadily increased throughout storage time. The highest rate of water losses during storage, as expressed by the drip loss, was observed in vacuum-packed ostrich muscles from 1.80% on day 4 to 3.62% on day 16 (*p* ≤ 0.05) as compared to the MAP1 and MAP2 packaging systems, where these values increased significantly in MAP1 from 1.43% on day 4 to 2.71% on the day 16, and in MAP2 from 1.61% on day 4 to 3.33% on the day 16. Moreover, only in case of the vacuum-packaging system were significant differences observed in the last period of the experimental storage, i.e., between day 12 and day 16.

Leygonie et al. [28] also noticed the increase in drip loss during storage time in frozen vacuum-packed ostrich meat stored for a month at −20 °C before thawing. However, Zakrys-Waliwander et al. [31], in their research on beef, found that drip loss was greater in MAP, but with a high oxygen level (80% of O_2_) relative to vacuum. Muscle tissue shows maximum water absorption and binding capacity immediately after slaughtering, which is related to its unchanged structure [32]. During vacuum packaging, the pressure generated in the intermicellar structures of the muscles increases the loss of water from the meat outside, thus increasing the drip loss [33]. As a consequence, the relatively high drip loss and slightly elevated pH during storage in the vacuum system negatively affect the processing quality of the ostrich meat.

### 2.4. Cooking Loss

Losses which occur during cooking depend on the storage; generally, the rate of drip of meat juice is higher due to increased storage time [34]. However, for this parameter, a significant increase (*p* ≤ 0.05) was observed only in the samples previously stored in the vacuum system on day 12 and 16 of refrigerated storage. This fact may be related to postmortem proteolysis, which leads to a weakening of the myofibrils that affect water distribution [35]. Similar tendencies were shown in the study conducted by Leygonie et al. [28].

### 2.5. WBSF

The initial WBSF value of ostrich meat in this study was 33.28 (N). The WBSF value decreased for all types of packaging during storage time (Table 2). Significant differences (*p* ≤ 0.05) were observed only in VP on day 12 and 16 of storage. In both MAP1 and MAP2, there were no significant changes in WBSF during storage, which could be caused by the presence of O_2_ in the packaging system. The higher oxygen content in packaging could increase protein aggregation and resulted in a lower proteolysis rate [36]. The higher value of WBSF in both packaging with modified atmosphere could be justified by a deceleration of proteolytic changes in muscles stored in the packaging with a modified atmosphere and higher oxygen concentration [37]. In relation to Destefanis et al. [38], who classified red meat into five groups of tenderness from very tender (WBSF < 32.96 N) to very hard (WBSF > 62.59 N), the ostrich meat samples in our study can be described as very tender after four days of storage, especially for the vacuum-packed samples (WBSF_VP_ = 30.39 N; WBSF_MAP1_ = 31.87 N; WBSF_MAP2_ = 31.77 N).

### 2.6. Microbial Quality

**Figure 1:**
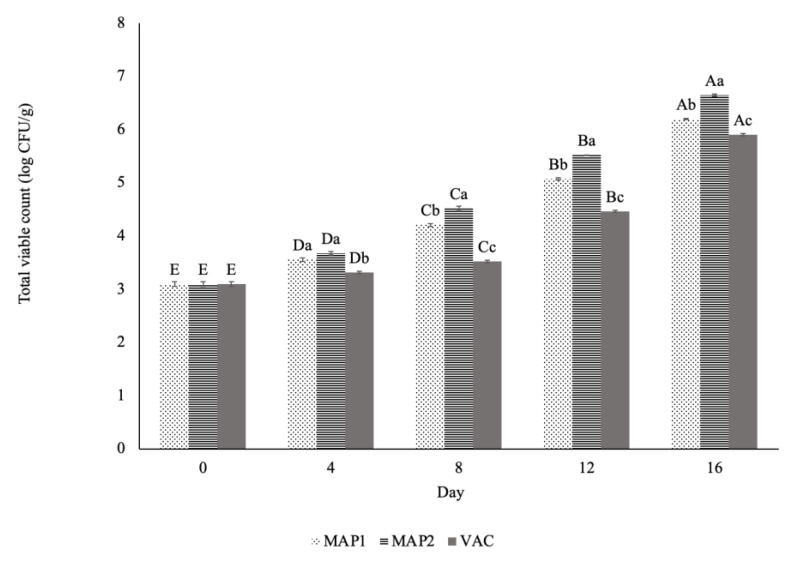
Total viable count (log CFU/g) in ostrich meat, as related to the type of packaging and refrigerated storage (mean value ± SEM). Mean values bearing different letters either between days (A–E) or between packaging systems (a–d) differ significantly at *p* ≤ 0.05.

Changes in the counts of total viable count (TVC) are demonstrated in Figure 1. The TVC in the ostrich muscle samples significantly increased (*p* ≤ 0.05) in each of the packaging methods during the experimental storage days. The lowest TVC was identified in VP, as compared to the two other methods. For the MAP2 packaging system, the TVC load was higher as compared to VP and MAP1, and on day 16 it increased to the level of 6.75 log CFU/g. The highest value of TVC in MAP2 can be associated with the highest concentration of the oxygen in this packaging system (60%), as compared to the other types of packaging. The relatively higher level of oxygen in this packaging system affected TVC growth, whereas CO_2_ had antimicrobial effects [22]. In another study on ostrich meat, Seydim et al. [16] also demonstrated that, after 10 days of storage, TVC growth was higher where the level of oxygen was 80% (air package system) in comparison with MAP with a lower concentration of O_2_. It should be noted that an EU report [39] also stated that modified atmosphere packaging (MAP) systems have proven to enhance product quality by inhibiting the growth of bacteria or of some pathogens.

A higher value of TVC in MAP packaging systems was obtained by Bingol et al. [40] during the storage of ostrich meat at 10 days. However, the initial TVC at day 0 in their investigations was higher (over 4 log CFU/g) as related to our study (3.1 log CFU/g), and the ostrich meat was stored at 4 °C compared to 2 °C in the current research. Ostrich carcasses had higher total viable counts of bacteria than beef carcasses, indicating more processing contamination for ostrich slaughter in a small abattoir. Sanitation and temperature were stated as being the most critical factors affecting the shelf life of products with or without modified atmosphere packaging conditions [41]. Currently, ostriches are mainly slaughtered in a special abattoir with EU certification. For example, ostrich meat produced in Poland is mostly exported to Western Europe and must fulfill special hygienic and sanitation requirements [20]. Moreover, as was mentioned by Alonso-Calleja et al. [42], Capita et al. [43] and Gonzalez-Montalvo [44], the relatively high microbial load recorded in ostrich meat in comparison with other red meats has been attributed to the high pH of the ostrich meat, which creates a good environment for fast microbial spoilage in some packaging systems.

## 3. Material and Methods

### 3.1. Samples and Packaging

Meat samples were obtained from the M. ilifibularis (IF) of 8 male ostriches, slaughtered at the age of 10–12 months, weighing from 90 to 95 kg. The slaughter procedure and carcass handling of the ostriches were described by Horbanczuk [45]. The IF muscle was excised (removal of external fat and visible connective tissue) from carcasses 24 h after slaughter and was cut, starting from the proximal side, into 2.5 cm thick steaks (sample weight: 150 g ± 15 g). Then, each group of 8 steaks was cut into three parts and assigned to one of three packaging systems (vacuum packaging and two conditions of modified atmosphere packaging).

Vacuum-packaging systems: Each meat sample was packed individually in PA/PE bags (thickness 90 μm (20/70), oxygen permeability 50 cm^3^/m^2^/24 h, CO_2_ permeability 140 cm^3^/m^2^/24 h, and water vapor permeability 6–8 g/m^2^/24 h) within 1 min after cutting and vacuum-packaged using a Vac-20SL2A packaging machine (Edesa Hostelera S.A., Barcelona, Spain). The in-package vacuum level was 2.5 kPa.

Modified atmosphere packaging (MAP) was carried out with two combinations of gases, O_2_:CO_2_:N_2_—40:40:20 (MAP1) and 60:30:10 (MAP2), respectively. The steaks were placed on PET/PE trays (parameters: 187 × 137 × 50 mm), and the film used was a 44 μm thick PET/CPP + AF laminate with maximum oxygen permeability not exceeding 10 cm^3^/m^2^/24 h/bar (EC04, Corenso, Helsinki, Finland). Meat samples were packed with an M3 packaging machine (Sealpack, Oldenburg, Germany).

The samples were stored in a refrigerator at 2 °C during the experiment for up to 16 days. Samples were collected in three independent replicates and analyzed at 0 (24 h after slaughter), 4, 8, 12 and 16 days of storage.

### 3.2. pH

The pH value of the muscles was measured in the middle part of each muscle, according to the PN-ISO 2917:1999 [46] standard. Results of the pH metric were obtained using a Testo 205 series pH meter equipped with a glass electrode, which was placed directly into the samples (2 cm deep into the steaks). Each measurement was performed in 6 repetitions, taking the mean value as the assay result. 

### 3.3. Color Parameters

The instrumental color analysis of ostrich meat was performed using a Minolta CR-400 chromometer calibrated against a white plate (L* = 98.45, a* = −0.10, b* = −0.13), using an 8 mm aperture illuminate D 65 (6500 K color temperature) at a standard observation of 2°. Meat color was expressed as: L* (lightness ranged from 0 to 100), a* (color axis ranged from greenness (−a*) to redness (+a*)), and b* (color axis ranged from blueness (−b*) to yellowness (+b*)). Color measurements of the steak were taken from each location, including every quarter and the centers of the surfaces. Data were collected directly after opening the package [16] under refrigerated conditions (2 ± 1 °C).

### 3.4. Drip Loss

Muscle weight loss during storage time was determined on the basis of the difference in weight before storage (M_0_) and after storage (M_1_). All samples were gently blotted with tissue paper prior to weighing. Drip loss (D_L_) was calculated using the equation:

$$D_{L}=\frac{\left(M_{0}-M_{1}\right)}{M_{0}}\times 100\%$$

### 3.5. Warner–Bratzler Shear Force Determination (WBSF)

After the respective storage time, at 0 (24 h after slaughter), 4, 8, 12 and 16 days of storage meat samples were prepared for shear force analysis according to Wyrwisz et al. [47]. The steaks (100 g ± 10 g) were cooked individually in closed PA/PE bags immersed in a water bath (Memmert, WNE 14, Schwabach, Germany) at 80 °C to achieve a final internal temperature of 73 °C, and then were subsequently cooled down in cold water and stored overnight at 2 ± 1 °C. Instrumental measurement of WBSF was conducted using a universal testing machine, Instron (Model 5965, Norwood, MA, USA), with a Warner–Bratzler shear attachment, consisting of a V-notch blade, according to Wyrwisz et al. [27]. The cores (1.27 cm in diameter and 2.5 ± 0.2 cm in length) were obtained from each steak, parallel to the muscle fiber’s orientation. A 500 N load cell was used, and the crosshead speed was set at 200 mm/min.

### 3.6. Cooking Loss

The percentage of cooking loss (CL) was determined through the measurement of sample mass before (M_i_) and after heat treatment (80 °C) after cooling to ambient temperature (M_f_). CL was calculated according to the equation:

$$\mathrm{CL}=\left(1-\frac{M_{f}}{M_{i}}\right)\times 100\%$$

### 3.7. Microbial Quality

Microbiological analysis of the ostrich meat was carried out every four days of storage, including total viable bacteria (TVC). Meat samples were taken from each packaging system and transported under continued refrigeration to an accredited laboratory where measurements were performed in triplicate in accordance with the PN-EN ISO 4833-1:2013-12 [48] standard for TVC; results were expressed as log10 CFU/g ostrich meat. The shelf life of the product was estimated on the basis of the limit of acceptability of 10^7^ bacteria/g according to ICMSF (1986) [49].

### 3.8. Statistical Analysis

A generalized linear mixed-model analysis (repeated measures ANOVA) was performed on all measured parameters including physical and microbial parameters in order to determine the fixed effect of packaging treatment and storage time as a repeated measure, as well as their interaction. Ostriches’ identity (bird number) was included in the model as a random factor. There were no outliers present in the dataset. Normality and homogeneity of residual variance assumptions were checked using the Shapiro test and examination of the normal plot, and these were met by all variables under investigation. PROC GLIMMIX of SAS v 9.4 (SAS Institute Inc., Cary, NC, USA) including the Tukey’s adjustment option was used to conduct the analysis. The validity of the models was tested using Akaike’s information criterion. The least square means for all significant effects in the models (*p* ≤ 0.05) were computed using the LSMEANS option. For all analyses, results are reported as means ± standard error of the mean (SEM).

## 4. Conclusions

The results from this study indicate that packaging systems including vacuum packaging (VP) and modified atmosphere packaging (MAP) using two combinations of gases: MAP1 (40% O_2_/40% CO_2_/20% N_2_) and MAP2 (60% O_2_/30% CO_2_/10% N_2_), and storage time had an influence on the physical features and microbial quality of ostrich meat, namely, pH, color (L*, a*, b*) and drip loss, which was the least in MAP1. Both the MAP1 and MAP2 systems affected color lightening and stabilizing redness (a*). The lowest total viable count of bacteria load was identified in VP, in comparison to MAP1 and MAP2. These data may help the ostrich meat industry to improve their packaging and storage operations while providing consumers with the highest quality ostrich meat products.

## Figures and Tables

**Table 1:** pH and color coordinates (L*, a* and b*) of ostrich meat, as related to the type of packaging and refrigerated storage (mean value ± SEM).

Parameter	Method	Day				
		0	4	8	12	16
pH	MAP1	5.99 ± 0.00 ^A^	5.90 ± 0.00 ^A,B^	5.89 ± 0.00 ^A,B,b^	5.85 ± 0.00 ^B,b^	5.81 ± 0.01 ^B,b^
	MAP2	5.99 ± 0.00	5.95 ± 0.00	5.92 ± 0.00 ^a,b^	5.91 ± 0.01 ^a,b^	5.93 ± 0.01 ^a,b^
	Vacuum	5.99 ± 0.00 ^B^	5.98 ± 0.00 ^B^	6.02 ± 0.01 ^A,b,a^	6.00 ± 0.01 ^A,B,a^	6.08 ± 0.00 ^A,a^
L*	MAP1	36.99 ± 0.12 ^B^	36.30 ± 0.15 ^B,b^	41.02 ± 0.49 ^A,a^	40.66 ± 0.19 ^A,a^	40.75 ± 0.10 ^A,a^
	MAP2	36.99 ± 0.12 ^C^	39.50 ± 0.07 ^B,a^	40.41 ± 0.07 ^B,a^	41.06 ± 0.07 ^A,a^	41.60 ± 0.11 ^A,a^
	Vacuum	36.99 ± 0.12 ^A,B^	37.39 ± 0.13 ^A,b^	36.85 ± 0.09 ^A,B,b^	36.29 ± 0.10 ^B,b^	34.22 ± 0.20 ^C,b^
a*	MAP1	19.97 ± 0.39 ^B^	21.13 ± 0.11 ^A,a^	20.73 ± 0.11 ^A,B,a^	20.56 ± 0.11 ^A,B,a^	20.50 ± 0.06 ^A,B,a^
	MAP2	19.97 ± 0.39 ^B^	21.75 ± 0.28 ^A,a^	20.74 ± 0.18 ^B,a^	20.74 ± 0.19 ^B,a^	20.64 ± 0.06 ^B,a^
	Vacuum	19.97 ± 0.39 ^A^	19.47 ± 0.16 ^A,B,b^	18.97 ± 0.21 ^B,b^	19.12 ± 0.26 ^A,B,b^	19.58 ± 0.21 ^A,B,b^
b*	MAP1	8.43 ± 0.07 ^B^	9.90 ± 0.21 ^A,a,b^	9.93 ± 0.08 ^A,a,b^	9.87 ± 0.07 ^A,a,b^	9.93 ± 0.05 ^A,a^
	MAP2	8.43 ± 0.07 ^B^	10.80 ± 0.05 ^A,a^	10.68 ± 0.04 ^A,a^	10.41 ± 0.10 ^A,a^	10.31 ± 0.07 ^A,a^
	Vacuum	8.43 ± 0.07	8.18 ± 0.08 ^b^	8.21 ± 0.12 ^b^	8.11 ± 0.14 ^b^	8.01 ± 0.03 ^b^

Mean values bearing different letters either for each day within rows (A, B, C) or between packaging systems within columns (a, b, c) differ significantly at *p* ≤ 0.05.

**Table 2:** Drip loss (%), cooking loss (%) and tenderness as a WBSF (N) in ostrich meat, as related to the type of packaging and refrigerated storage (mean value ± SEM).

Parameter	Method	Day				
		0	4	8	12	16
Drip loss	MAP1	-	1.43 ± 0.01 ^B,c^	1.65 ± 0.01 ^B,c^	2.21 ± 0.01 ^A,B,c^	2.71 ± 0.00 ^A,c^
	MAP2	-	1.61 ± 0.00 ^B,b^	2.01 ± 0.01 ^A,B,b^	2.50 ± 0.00 ^A,b,b^	3.33 ± 0.01 ^A,b^
	Vacuum	-	1.80 ± 0.02 ^C,a^	2.18 ± 0.03 ^B,C,a^	2.60 ± 0.03 ^B,a^	3.62 ± 0.01 ^A,a^
Cooking loss	MAP1	32.95 ± 0.24	34.48 ± 0.23	35.51 ± 0.11	36.10 ± 0.01	36.31 ± 0.05
	MAP2	32.95 ± 0.24	34.73 ± 0.01	35.64 ± 0.02	36.23 ± 0.20	36.35 ± 0.04
	Vacuum	32.95 ± 0.24 ^B^	35.02 ± 0.11 ^A,B^	36.21 ± 0.29 ^A,B^	36.65 ± 0.18 ^A^	37.61 ± 0.39 ^A^
WBSF	MAP1	33.28 ± 0.32	31.87 ± 0.18	31.40 ± 0.19	30.92 ± 0.22 ^a^	30.22 ± 0.03 ^a^
	MAP2	33.28 ± 0.32	31.77 ± 0.15	31.66 ± 0.30	31.29 ± 0.17 ^a^	31.07 ± 0.25 ^a^
	Vacuum	33.28 ± 0.32 ^A^	30.39 ± 0.14 ^B^	30.29 ± 0.08 ^B^	28.59 ± 0.15 ^C,b^	28.38 ± 0.08 ^C,b^

Mean values bearing different letters either for each day within rows (A, B, C) or between packaging systems within columns (a, b, c) differ significantly at *p* ≤ 0.05.

## Data Availability

Not applicable.

## Sample Availability

Samples of the compounds are not available from the authors.
